# Effect of prior thyroid cancer on survival of primary liver cancer: a study based on the SEER database

**DOI:** 10.1038/s41598-022-17729-4

**Published:** 2022-08-16

**Authors:** Hong Liu, Xin Heng, Yuan Tian, Zhongming Yang

**Affiliations:** Department of Oncology and Hematology, The Affiliated Traditional Chinese Medicine Hospital of Southwest Medical University, 182 Chunhui Road, Longmatan District, Luzhou, 646000 People’s Republic of China

**Keywords:** Cancer, Cancer, Oncology

## Abstract

To explore the effect of prior thyroid cancer on the survival of primary liver cancer (PLC). Eligible PLC patients were selected from the Surveillance, Epidemiology, and End Results (SEER) database during 2004–2016. Propensity score matching (PSM) was used to create a highly comparable control group that PLC patients without prior thyroid cancer. All PLC patients were divided into three groups based on the survival information: (1) PLC-specific death; (2) death due to other causes; (3) alive. The effect sizes were presented by the corresponding hazard ratio (HR) and 95% confidence intervals (CI). Totally, 142 PLC patients with prior thyroid cancer and 1420 PLC patients without prior thyroid cancer were included. During the follow-up period, 714 (45.71%) PLC patients died of liver cancer while 638 (40.85%) PLC patients were alive. Median survival time for PLC patients was 11.00 months, respectively. PLC patients with prior thyroid cancer have a lower risk of death (HR = 0.64; 95% CI: 0.48–0.86). Subgroup analyses stratified by gender displayed the similar relation in female patients with PLC. Prior thyroid cancer may be a protective factor for liver cancer death in PLC patients, especially in female patients.

## Introduction

Primary liver cancer (PLC) is the fourth most prevalent leading cause of cancer-related death around the world^1–3^. Despite enormous progress in the modern therapeutic era, 5-year net survival of PLC ranging from 5 to 30%^4^. Over the past 20 years, liver cancer has been the tumor with the greatest increased mortality^5^. By 2030, the number of liver cancer cases worldwide could rise to 1 million^6^. The steadily increased incidence and high mortality rates have aroused people’s attention^7^.

With the development of cancer therapy and an increase in the rates of early diagnosis, the number of long-term cancer survivors continues to rise^8^. Better survival expectations increase the possibility of developing a second primary malignancy among survivors of cancers^9,10^. There is a pressing need to understand the clinical characteristics and survival outcomes of these populations. Several studies have reported that prior cancer history appeared to have an impact on second primary cancer survival^11–13^. Thyroid cancer, as cancer with a high survival rate, its influence on primary cancer survival has also been discussed^14–16^. Thyroid cancer has been found a benefit to the survival of ovarian^16^ and breast cancer^15^. The results of the previous study have encouraged the exploration of the effect of thyroid cancer on other cancers. A study by Mantovani et al. demonstrated that hypothyroidism was associated with a severe degree of nonalcoholic fatty liver disease (NAFLD)^17^. Furthermore, decreased thyroid function was related to a variety of metabolic abnormalities, such as insulin resistance, dyslipidemia, and lipid peroxidation, which all may increase the risk of liver damage^18^. Abnormal thyroid function may have an adverse effect on liver cancer mortality^19^. The association between prior thyroid cancer and the survival of PLC need to be explored.

Herein, we used data from the Surveillance, Epidemiology, and End Results (SEER) database to analyze the survival of PLC patients with or without a prior thyroid cancer. We conducted this study to determine whether prior thyroid cancer affects the survival of PLC patients.

## Materials and methods

### Study design and population

In this retrospective cohort study, we selected patients with PLC (the *International Classification of Diseases for Oncology*, Third Edition [ICD-O-3] code: C22.0, liver; C22.1, intrahepatic bile duct) from the SEER database during 2004 and 2016 based on November 2018 submission. SEER database provides complete patient data abstracted from 18 geographically diverse populations that represent rural, urban, and regional populations. Excluding criteria were as follows: (1) patients with a history of liver cancer; (2) patients demographic information lost; (3) < 18 years old; (4) patients whose follow-up time was 0 or lost to follow-up. Follow-up time for each patient was calculated from the date of cohort entry to the date of death or the end of follow-up (31 December 2016), and patients were followed up once a month. As all SEER data were accessed with approval from the SEER database, this article does not contain any studies with human participants or animals performed by any of the authors.

### Data selection

We gathered variables of age, gender (female or male), race (white; black; others), marital status [married (including common law) or not married], tumor size (less than 5 cm; greater than or equal to 5 cm; unknown), regional nodes positive (yes; no; unknown), primary site (ICD-O-3, code C22.0 or C22.1), radiation therapy (none or unknown; yes), surgery (none or unknown; surgery performed), chemotherapy (none or unknown; yes), histologic grade (grade I, well differentiated; grade II, moderately differentiated; grade III, poorly differentiated; grade IV, undifferentiated; unknown), American Joint Committee on Cancer (AJCC) T status (T1; T2; T3; T4; unknown), AJCC N status (N0; N1; unknown), AJCC M status (M0; M1; unknown), SEER historic stage (localized; regional; distant; unknown), survival months, vital status (alive or dead), SEER cause death classification (alive; death due to liver cancer; death due to other cause).

### Variable definitions

The primary site code (C22.0, liver) and the ICD-O-3 histology codes were used to identify cases with hepatocellular Carcinoma: from 8170 to 8175. The primary site code (C22.0, liver) and the ICD-O-3 histology codes of cases with intrahepatic cholangiocarcinoma was 8160. Prior thyroid cancer was defined as being diagnosed with thyroid cancer (the ICD-O-3 primary site code: C73.9, thyroid gland) before patients were diagnosed with liver cancers.

The eligible patients’ survival status was divided into three groups: (1) PLC-specific death; (2) death due to other causes; (3) alive.

### Statistical analysis

Propensity Score Matching (PSM) is a commonly used statistical method that establishes a new control group by discarding outlier control subjects so that reduce the unwanted influences of covariates as well as measures the intended variable properly^20^. We used PSM with a caliper value of 0.1 and a ratio of 1:10 for matching, i.e. each patient with prior thyroid cancer was matched by age and gender with 10 patients without prior thyroid cancer (Supplementary Table 1).

Kolmogorov–Smirnov normality test was used for the measurement data. And measurement data of normal distribution was described as mean ± standard deviation (mean ± SD), while the measurement data of non-normal distribution were presented as median and quartile [M (Q1, Q3)]. And an independent-sample *t* test and Mann–Whitney *U* rank-sum test were conducted, respectively. The enumeration data were manifested as cases and the constituent ratio [n (%)] and were compared using the chi-square test or the Fisher’s exact test.

Cox proportional hazard model was used to explore the effect of prior thyroid cancer on the risk of PLC-specific death. And the product-limit (KM) method was used to plot the cumulative incidence function curve. In the competing risk model, the prior thyroid cancer was taken as the main variable, survival month and survival status (i.e. PLC-specific death; other cause-specific death; alive) were used as the outcome variables. Model 1 was undusted model. Model 2 was adjusted for age and gender, and Model 3 was adjusted for all confounding factors, i.e. age, gender, race, marital status, tumor size, regional nodes positive, primary site, histology groupings, radiation therapy, surgery, chemotherapy, histologic grade, AJCC T status, AJCC N status, AJCC M status, SEER historic stage.

SAS software (SAS Institute Inc., Cary, NC, USA; version 9.4) was used for all analyses. All the statistical tests were two-sided, and *P* < 0.05 was considered to be statistically significant.

### Ethics declarations

Our data are from SEER database. Since all subjects in the database were anonymous, informed consent and ethical approval were not required. All methods were carried out in accordance with relevant guidelines and regulations.

## Results

### Baseline information of participants

**Figure 1:**
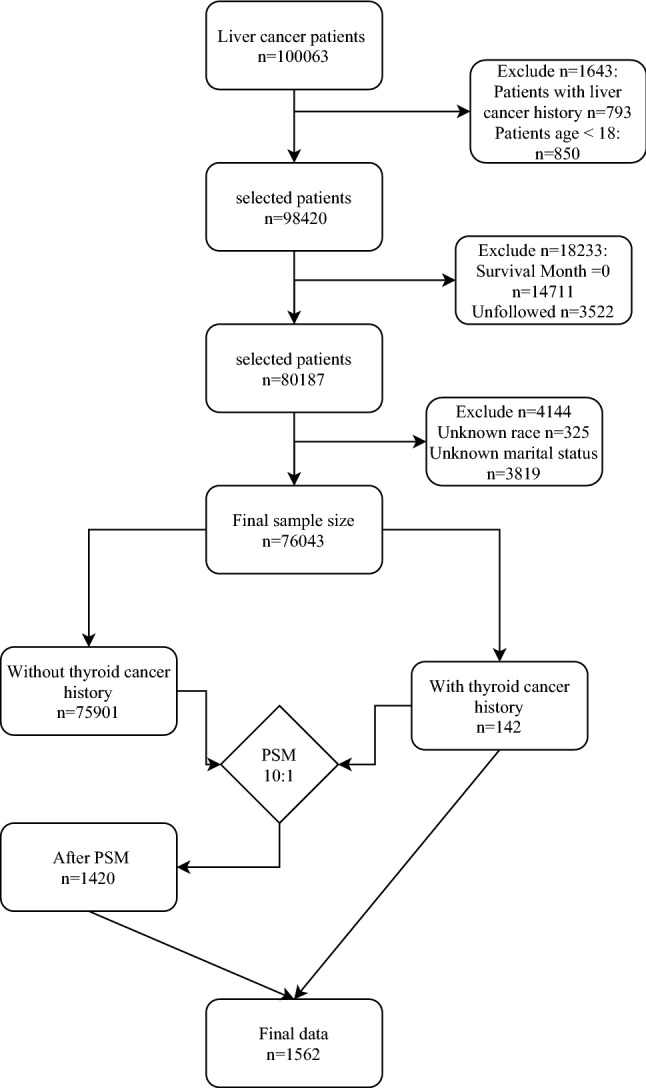
Flow chart of the study participants.

We originally selected 100,063 patients with liver cancer from the SEER database, 793 patients with a history of liver cancer, 850 patients whose ages were younger than 18 years old, 14,711 patients whose survival months were zero, 3522 patients who were lost follow-up, 4144 patients with unknown demographic information were excluded. Finally, a total of 76,043 patients were included in this study, among them, 1420 PLC patients without prior thyroid cancer and 142 PLC patients with prior thyroid cancer after PSM matching processing. The characteristics of the eligible patients before PSM matching processing are shown in Supplementary Table 2. From January 1, 2004, to December 31, 2016, the loss rate of follow-up in this study was 4%. The patient’s selection strategy is present in Fig. 1.

The average age of patients was 65.45 ± 10.83 years old, and the majority of patients were males (1052, 67.35%), white race (1029, 65.88%), and married (827, 52.94%). Comparing PLC without prior thyroid cancer, PLC patients who had prior thyroid cancer had longer survival months (*P* < 0.001). And PLC patients with prior thyroid cancer had higher proportion of females (*P* < 0.001), white race (*P* = 0.039), dead (*P* = 0.020), and death due to other causes (*P* < 0.001) than those who did not have thyroid cancer before. The main characteristics of the eligible patients are illustrated in Table 1.

**Table 1:** Characteristics of the sample population.

Variable	Total	Without prior thyroid cancer (n = 1420)	With prior thyroid cancer (n = 142)	Statistics	*P*-value
Age, mean ± SD	65.45 ± 10.83	65.45 ± 10.71	65.49 ± 12.04	t = -0.04	0.967
**Gender, n (%)**				χ^2^ = 23.152	< 0.001
Female	510 (32.65)	438 (30.85)	72 (50.70)		
Male	1052 (67.35)	982 (69.15)	70 (49.30)		
**Race, n (%)**				χ^2^ = 6.513	0.039
White	1029 (65.88)	924 (65.07)	105 (73.94)		
Black	163 (10.44)	156 (10.99)	7 (4.93)		
Others	370 (23.69)	340 (23.94)	30 (21.13)		
**Marital status, n (%)**				χ^2^ = 3.639	0.056
Married	827 (52.94)	741 (52.18)	86 (60.56)		
Not married	735 (47.06)	679 (47.82)	56 (39.44)		
**Tumor size, n (%)**				χ^2^ = 10.555	0.005
< 5 cm	696 (44.56)	634 (44.65)	62 (43.66)		
≥ 5 cm	538 (34.44)	475 (33.45)	63 (44.37)		
Unknown	328 (21.00)	311 (21.90)	17 (11.97)		
**Regional nodes positive, n (%)**				χ^2^ = 7.377	0.025
Yes	21 (1.34)	18 (1.27)	3 (2.11)		
No	54 (3.46)	43 (3.03)	11 (7.75)		
Unknown	1487 (95.20)	1359 (95.70)	128 (90.14)		
**The primary site, n (%)**				χ^2^ = 3.915	0.048
C22.0-Liver	1387 (88.80)	1268 (89.30)	119 (83.80)		
C22.1-Intrahepatic bile duct	175 (11.20)	152 (10.70)	23 (16.20)		
**Radiation therapy, n (%)**				χ^2^ = 8.556	0.003
None / Unknown	1479 (94.69)	1352 (95.21)	127 (89.44)		
Yes	83 (5.31)	68 (4.79)	15 (10.56)		
**Surgery, n (%)**				χ^2^ = 11.196	< 0.001
None / Unknown	1152 (73.75)	1064 (74.93)	88 (61.97)		
Surgery performed	410 (26.25)	356 (25.07)	54 (38.03)		
**Chemotherapy, n (%)**				χ^2^ = 0.287	0.592
None / Unknown	913 (58.45)	827 (58.24)	86 (60.56)		
Yes	649 (41.55)	593 (41.76)	56 (39.44)		
**Histologic grade, n (%)**				Fisher	< 0.001
Grade I	130 (8.32)	107 (7.54)	23 (16.20)		
Grade II	292 (18.69)	259 (18.24)	33 (23.24)		
Grade III	136 (8.71)	119 (8.38)	17 (11.97)		
Grade IV	9 (0.58)	8 (0.56)	1 (0.70)		
Unknown	995 (63.70)	927 (65.28)	68 (47.89)		
**AJCC T status, n (%)**				χ^2^ = 12.204	0.016
T1	533 (34.12)	469 (33.03)	64 (45.07)		
T2	262 (16.77)	240 (16.90)	22 (15.49)		
T3	264 (16.90)	242 (17.04)	22 (15.49)		
T4	63 (4.03)	55 (3.87)	8 (5.63)		
Unknown	440 (28.17)	414 (29.15)	26 (18.31)		
**AJCC N status, n (%)**				χ^2^ = 9.210	0.010
N0	1071 (68.57)	964 (67.89)	107 (75.35)		
N1	107 (6.85)	93 (6.55)	14 (9.86)		
Unknown	384 (24.58)	363 (25.56)	21 (14.79)		
**AJCC M status, n (%)**				χ^2^ = 7.562	0.023
M0	1102 (70.55)	997 (70.21)	105 (73.94)		
M1	177 (11.33)	155 (10.92)	22 (15.49)		
Unknown	283 (18.12)	268 (18.87)	15 (10.56)		
**SEER historic stage, n (%)**				χ^2^ = 16.737	< 0.001
Localized	672 (43.02)	599 (42.18)	73 (51.41)		
Regional	367 (23.50)	341 (24.01)	26 (18.31)		
Distant	174 (11.14)	149 (10.49)	25 (17.61)		
Unknown	349 (22.34)	331 (23.31)	18 (12.68)		
Survival month, M (Q_1_,Q_3_)	11.00 (4.00,20.00)	11.00 (4.00,19.00)	14.00 (7.00,39.00)	Z = 4.959	< 0.001
**Vital status, n (%)**				χ^2^ = 5.418	0.020
Alive	638 (40.85)	593 (41.76)	45 (31.69)		
Dead	924 (59.15)	827 (58.24)	97 (68.31)		
**SEER cause death classification, n (%)**				χ^2^ = 24.399	< 0.001
Alive	638 (40.85)	593 (41.76)	45 (31.69)		
Death due to liver cancer	714 (45.71)	655 (46.13)	59 (41.55)		
Death due to other cause	210 (13.44)	172 (12.11)	38 (26.76)		

*AJCC* American Joint Committee on Cancer, *SEER* the Surveillance, Epidemiology, and End Results.

### Confounding factors

Variables including age, tumor size, regional nodes positive, primary site, histology groupings, surgery, chemotherapy, histologic grade, AJCC T status, AJCC N status, AJCC M status, and SEER historic stage all showed significant association with the risk of PLC-specific death were included in competing risk model (Table 2). Gender was included in the model as a variable that was often adjusted. Considering that marital status may be related to outcome variables, it was also included in the model in our study.

**Table 2:** Screening for potential confounding factors.

Variable	HR (95% CI)	*P*-value
Age	1.02 (1.01–1.02)	< 0.001
Gender (female)	1.11 (0.95–1.30)	0.175
**Race**		
White	Ref	
Black	0.98 (0.76–1.26)	0.870
Other	1.02 (0.86–1.21)	0.860
**Marital status**		
Not married	Ref	
Married	0.90 (0.78–1.04)	0.143
**Tumor size**		
< 5 cm	Ref	
≥ 5 cm	2.78 (2.32–3.33)	< 0.001
Unknown	4.37 (3.60–5.31)	< 0.001
**Regional nodes positive**		
No	Ref	
Yes	2.83 (1.26–6.36)	0.012
Unknown	2.66 (1.43–4.96)	0.002
**Primary site**		
C22.1-Intrahepatic bile duct	Ref	
C22.0-Liver	0.47 (0.38–0.58)	< 0.001
Radiation therapy (none/unknown)	1.31 (0.93–1.85)	0.118
Surgery (none/unknown)	4.33 (3.44–5.45)	< 0.001
Chemotherapy (none/unknown)	1.42 (1.23–1.64)	< 0.001
**Histologic grade**		
Grade I	Ref	
Grade II	1.33 (0.91–1.93)	0.137
Grade III	2.73 (1.84–4.07)	< 0.001
Grade IV	4.76 (1.90–11.94)	< 0.001
Unknown	2.36 (1.69–3.30)	< 0.001
**AJCC T status**		
T1	Ref	
T2	1.24 (0.97–1.60)	0.085
T3	3.64 (2.92–4.54)	< 0.001
T4	4.28 (3.03–6.03)	< 0.001
Unknown	3.35 (2.73–4.12)	< 0.001
**AJCC N status**		
N0	Ref	
N1	2.65 (2.10–3.35)	< 0.001
Unknown	2.14 (1.82–2.52)	< 0.001
**AJCC M status**		
M0	Ref	
M1	3.67 (3.02–4.46)	< 0.001
Unknown	2.30 (1.91–2.76)	< 0.001
**SEER historic stage**		
Localized	Ref	
Regional	2.22 (1.82–2.70)	< 0.001
Distant	5.40 (4.30–6.58)	< 0.001
Unknown	3.19 (2.61–3.88)	< 0.001

*HR* hazard ratio, *CI* confidence interval, *Ref* reference, *AJCC* American Joint Committee on Cancer, *SEER* the Surveillance, Epidemiology, and End Results.

### The effect of prior thyroid cancer on the survival of PLC patients

**Figure 2:**
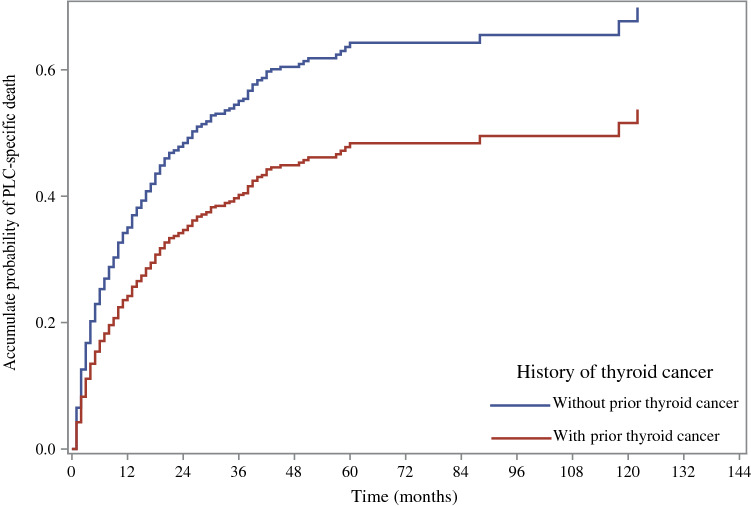
The cumulative incidence of PLC-specific death in PLC patients with and without prior thyroid cancer. *PLC* primary liver cancer.

The cumulative incidence of PLC-specific death in PLC patients with and without prior thyroid cancer are shown in Fig. 2. The result showed that PLC patients with prior thyroid cancer had a lower risk of death from liver cancer.

Supplementary Table 3 showed that PLC patients who had a thyroid cancer history had a decreased PLC-specific death [hazard ratio (HR) = 0.618; 95% confidence interval (CI): 0.477–0.801, *P* < 0.001]. Compared with PLC patients who did not have prior thyroid cancer, the risk of death from liver cancer was reduced by 0.37 folds in patients with prior thyroid cancer [hazard ratio (HR) = 0.63; 95% confidence interval (CI): 0.50–0.81, *P* < 0.001]. After adjusting the age and gender, the risk of death from liver cancer was decreased by 0.39 folds in patients who had prior thyroid cancer (HR = 0.61; 95% CI: 0.48–0.79, *P* < 0.001). When all the confounders were adjusted, among patients with prior thyroid cancer, the risk of death from liver cancer was significantly lower than those without (HR = 0.64; 95% CI: 0.48–0.86, *P* = 0.003). Thus, prior thyroid cancer may be a protective factor for liver cancer death in patients with PLC (Table 3).

**Table 3:** The effect of thyroid cancer history on the risk of PLC-specific death among PLC patients.

Variable	Model 1^a^		Model 2^b^		Model 3^c^	
	HR (95% CI)	*P-*value	HR (95% CI)	*P-*value	HR (95% CI)	*P-*value
Thyroid cancer history	0.63 (0.50–0.81)	< 0.001	0.61 (0.48–0.79)	< 0.001	0.64 (0.48–0.86)	0.003

*PLC* primary liver cancer, *HR* hazard ratio, *CI* confidence interval.

^a^Model 1 was established as a crude model without adjustment.

^b^Model 2 adjusted for age and gender.

^c^Model 3 adjusted for age, gender, race, marital status, tumor size, regional nodes positive, primary site, histology groupings, surgery, chemotherapy, histologic grade, AJCC T status, AJCC N status, AJCC M status, SEER historic stage.

In the sensitivity analysis, the result before PSM treatment (Supplement Table 2) was similar to that after treatment (Table 3), i.e. prior thyroid cancer was related to the decreased risk of PLC-specific death in patients with PLC.

### Association of prior thyroid cancer with the survival of PLC patients based on gender grouping

**Figure 3:**
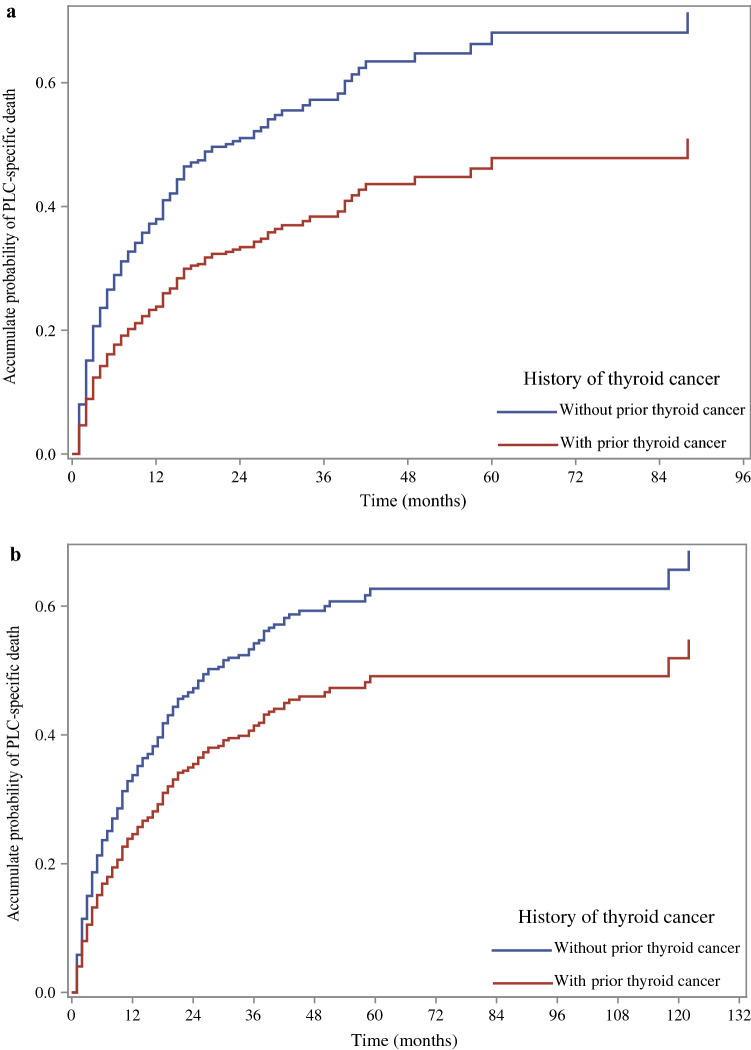
The cumulative incidence of PLC-specific death between PLC patients with and without prior thyroid cancer in female (**a**) and male (**b**). *PLC* primary liver cancer.

Among gender grouping, female patients with prior thyroid cancer had a lower risk of PLC death (HR = 0.49; 95% CI: 0.34–0.71, *P* < 0.001). In Model 2, this difference remained significant in the subgroups mentioned above. In Model 3, the potential protective effect of prior thyroid cancer was only found in female patients with PLC (HR = 0.53; 95% CI: 0.32–0.90, *P* = 0.018) (Table 4). The cumulative incidence of PLC-specific death between PLC patients with and without prior thyroid cancer in females is depicted in Fig. 3a. The cumulative incidence of PLC-specific death between PLC patients with and without prior thyroid cancer in males is displayed in Fig. 3b.

**Table 4:** Association of prior thyroid cancer with the risk of PLC-specific death among PLC patients by gender.

Subgroup	Variable	Model 1^a^		Model 2^b^		Model 3^c^	
		HR (95% CI)	*P-*value	HR (95% CI)	*P-*value	HR (95% CI)	*P-*value
Gender	Male	0.74 (0.51–1.08)	0.118	0.73 (0.50–1.06)	0.100	0.79 (0.51–1.23)	0.299
	Female	0.49 (0.34–0.71)	< 0.001	0.51 (0.35–0.73)	< 0.001	0.53 (0.32–0.90)	0.018

*PLC* primary liver cancer, *HR* hazard ratio, *CI* confidence interval.

^a^Model 1 was established as a crude model without adjustment.

^b^Model 2 adjusted for age (or and gender).

^c^Model 3 adjusted for age, (or and gender), race, marital status, tumor size, regional nodes positive, primary site, histology groupings, surgery, chemotherapy, histologic grade, AJCC T status, AJCC N status, AJCC M status, (or and SEER historic stage).

## Discussion

An increase in cancer survivors has recently been observed due to advances in diagnosis and treatment strategies. With longer survival, patients are more likely to be subsequently diagnosed with second primary malignancy. It is important to determine the clinical significance and survival outcome of previous malignancies, as these factors may influence clinical trial design, oncology practice, and treatment decisions. Hypothyroidism has recently been identified as a predisposing factor for the development of liver cancer^21^. However, the role of thyroid cancer in PLC remains unclear. The present study evaluated the relationship between thyroid cancer and liver cancer mortality in a seer database consisting of middle-aged 1562 subjects. The result of this study indicated that among PLC patients, especially in female patients, prior thyroid cancer reduces the risk of PLC-specific death.

Our study observed that prior thyroid cancer may be a protective factor for liver cancer death. Thyroid cancer patients would suffer from hypothyroidism which is a common condition of thyroid hormone (TH) deficiency^22^ after undergoing a series of treatments to damage the diseased thyroid gland, such as surgery and the use of radioiodine^23^. While hypothyroidism is associated with poorer overall and recurrence-free survival of hepatocellular carcinoma patients receiving liver transplantation^24^. Pinter et al. observed that thyroid-stimulating hormone (TSH) and free tetraiodothyronine (FT4) were associated with prognostic factors of hepatocellular carcinoma^3^. A study by Kowalik et al. suggests that re-activation of the thyroid hormone triiodothyronine (T3)/thyroid hormone receptor β (TRβ) axis induces differentiation of neoplastic cells towards a more benign phenotype and that T3 or its analogues, particularly agonists of the TRβ, can represent useful tools in liver cancer therapy. In addition, T3 was shown to increase mitochondrial function and respiration in hepatocellular carcinoma cells^25^. The protective effect of prior thyroid cancer on PLC survival may be explained by the effects of TH on PLC. Changes in dietary patterns after the first primary cancer may also lead to better survival with the second primary cancer. Cancer patients tend to have good eating habits after being diagnosed, such as reducing alcohol consumption^26^. Previous studies speculated that a history of alcohol consumption may be a potential prognostic factor for liver cancer patients^27,28^.

In addition, the intensified medical surveillance after a first primary cancer leads to earlier detection of second primary cancers^29^. Early detection can effectively improve the prognosis of cancer patients and reduce the mortality rate^1^. A study by Huang et al. supported that increased surveillance after a first primary cancer leads to better outcomes for second primary cancers^30^. The first primary cancer may guide the frequency of second primary malignancy surveillance, which may lead to early diagnosis and better survival outcomes.

In this study, prior thyroid cancer may be a protective factor for PLC death in female patients rather than male patients. Thyroid cancer is reported to be female predominant while male patients have more aggressive behaviors and worse prognosis compared with female^31^. Liver metastases from thyroid cancer were more frequently diagnosed in male patients^32^. Some studies showed that estrogens may play a role in favoring the malignant progression of thyroid tissue to cancer^33,34^. We speculate that the better prognosis in female patients may be the reflection of the higher aggressive biology and mortality of thyroid cancer in male.

We conducted a retrospective cohort study based on the SEER database during 2004–2016, and found that prior thyroid cancer may decrease the risk of death for PLC. This finding suggests that prior cancers may influence the risk of subsequent cancers’ specific death, and researchers and clinicians should pay more attention to the possible impact of disease history on the results of their research or diagnosis. Our research has the following advantages. First of all, large-scale information from the SEER database is the highlights of our study. Moreover, the SEER database has high data accuracy. Furthermore, to our knowledge, it is the first attempt to explore the effect of prior thyroid cancer on the survival of PLC. However, the following limitations should be taken into consideration. Some information (such as the use of TH replacement drugs) was not investigated in the SEER database, so there may be some potential confounding factors that had not been considered. To reduce the impact of these limitations, clinical trials with a larger sample size should be conducted to verify our results.

## Conclusion

Prior thyroid cancer may reduce the risk of PLC-specific death among PLC patients, especially female patients. It means that prior thyroid cancers may have an important effect on the development of PLC, and researchers and clinicians should pay more attention to the potential impact of prior thyroid cancer on their research or diagnosis related to PLC.

## Supplementary Information

Supplementary Tables.

## Data Availability

The datasets used and/or analyzed during the current study are available from the corresponding author on reasonable request.
