# Supplementary material for: Effect of prior thyroid cancer on survival of primary liver cancer: a study based on the SEER database
Source: Sci Rep. 2022 Aug 16;12:13887. doi: 10.1038/s41598-022-17729-4 (PMC9381514; doi:10.1038/s41598-022-17729-4)
Supplement: Supplementary file 1 — Supplementary Tables. [file 41598_2022_17729_MOESM1_ESM.docx]

**Supplement Table 1 PSM process by age and gender of PLC patients without prior thyroid cancer**

| **Variable** | **Before PSM** | | | |  | **After PSM** | | | |
| --- | --- | --- | --- | --- | --- | --- | --- | --- | --- |
|  | **Prior thyroid cancer** | | **Statistics** | ***P*-value** |  | **Prior thyroid cancer** | | **Statistics** | ***P*-value** |
|  | **Without**  **(n= 75901)** | **With**  **(n=142)** |  |  |  | **Without (n=1420)** | **With**  **(n=142)** |  |  |
| Gender, n (%) |  |  | χ^2^=35.446 | <0.001 |  |  |  | χ^2^=23.152 | <.001 |
| Female | 21395 (28.19) | 72 (50.70) |  |  |  | 438 (30.85) | 72 (50.70) |  |  |
| Male | 54506 (71.81) | 70 (49.30) |  |  |  | 982 (69.15) | 70 (49.30) |  |  |
| Age, Mean±SD | 64.24 ± 11.35 | 65.49 ± 12.04 | t=1.31 | 0.190 |  | 65.45 ± 10.71 | 65.49 ± 12.04 | t=-0.04 | 0.967 |

Abbreviations: PSM, propensity score matching.

**Supplementary Table 2 Characteristics of the eligible patients** **before PSM matching processing**

| **Variables** | **Total (n=76043)** | **Without prior thyroid cancer (n=75901)** | **With prior thyroid cancer (n=142)** | **Statistics** | ***P*-value** | | |
| --- | --- | --- | --- | --- | --- | --- | --- |
| Age, Mean±SD | 64.25 ± 11.35 | 64.24 ± 11.35 | 65.49 ± 12.04 | t=-1.31 | 0.190 | |  |
| Sex, n (%) |  |  |  | χ^2^=35.466 | <0.001 | |  |
| Female | 21467 (28.23) | 21395 (28.19) | 72 (50.70) |  |  | |  |
| Male | 54576 (71.77) | 54506 (71.81) | 70 (49.30) |  |  | |  |
| Race, n (%) |  |  |  | χ^2^=8.145 | 0.017 | |  |
| Black | 9422 (12.39) | 9415 (12.40) | 7 (4.93) |  |  | |  |
| Others | 12807 (16.84) | 12777 (16.83) | 30 (21.13) |  |  |  |  |
| White | 53814 (70.77) | 53709 (70.76) | 105 (73.94) |  |  |  |  |
| Marital status, n (%) |  |  |  | χ^2^=2.288 | 0.130 |  |  |
| Married | 41250 (54.25) | 41164 (54.23) | 86 (60.56) |  |  |  |  |
| Not married | 34793 (45.75) | 34737 (45.77) | 56 (39.44) |  |  |  |  |
| Tumor size, n (%) |  |  |  | χ^2^=8.989 | 0.011 |  |  |
| <5 cm | 30961 (40.72) | 30899 (40.71) | 62 (43.66) |  |  |  |  |
| ≥5 cm | 28225 (37.12) | 28162 (37.10) | 63 (44.37) |  |  |  |  |
| Unknown | 16857 (22.17) | 16840 (22.19) | 17 (11.97) |  |  |  |  |
| Regional nodes positive, n (%) |  |  |  | χ^2^=5.280 | 0.071 |  |  |
| No | 2963 (3.90) | 2952 (3.89) | 11 (7.75) |  |  |  |  |
| Yes | 934 (1.23) | 931 (1.23) | 3 (2.11) |  |  |  |  |
| Unknown | 72146 (94.88) | 72018 (94.88) | 128 (90.14) |  |  |  |  |
| The primary site, n (%) |  |  |  | χ^2^=4.629 | 0.031 |  |  |
| C22.0-Liver | 67955 (89.36) | 67836 (89.37) | 119 (83.80) |  |  |  |  |
| C22.1-Intrahepatic bile duct | 8088 (10.64) | 8065 (10.63) | 23 (16.20) |  |  |  |  |
| Radiation therapy, n (%) |  |  |  | χ^2^=0.536 | 0.464 |  |  |
| None / Unknown | 69334 (91.18) | 69207 (91.18) | 127 (89.44) |  |  |  |  |
| Yes | 6709 (8.82) | 6694 (8.82) | 15 (10.56) |  |  |  |  |
| Surgery, n (%) |  |  |  | χ^2^=12.328 | <0.001 |  |  |
| None / Unknown | 56849 (74.76) | 56761 (74.78) | 88 (61.97) |  |  |  |  |
| Surgery performed | 19194 (25.24) | 19140 (25.22) | 54 (38.03) |  |  |  |  |
| Chemotherapy, n (%) |  |  |  | χ^2^=0.699 | 0.403 |  |  |
| None / Unknown | 43416 (57.09) | 43330 (57.09) | 86 (60.56) |  |  |  |  |
| Yes | 32627 (42.91) | 32571 (42.91) | 56 (39.44) |  |  |  |  |
| Histologic grade, n (%) |  |  |  | χ^2^=15.022 | 0.005 |  |  |
| Grade II | 12303 (16.18) | 12270 (16.17) | 33 (23.24) |  |  |  |  |
| Grade III | 6992 (9.19) | 6975 (9.19) | 17 (11.97) |  |  |  |  |
| Grade IV | 585 (0.77) | 584 (0.77) | 1 (0.70) |  |  |  |  |
| Unknown | 48321 (63.54) | 48253 (63.57) | 68 (47.89) |  |  |  |  |
| Grade I | 7842 (10.31) | 7819 (10.30) | 23 (16.20) |  |  |  |  |
| AJCC T status, n (%) |  |  |  | χ^2^=10.934 | 0.053 |  |  |
| T1 | 25745 (33.86) | 25681 (33.83) | 64 (45.07) |  |  |  |  |
| T2 | 13213 (17.38) | 13191 (17.38) | 22 (15.49) |  |  |  |  |
| T3 | 13996 (18.41) | 13974 (18.41) | 22 (15.49) |  |  |  |  |
| T4 | 2980 (3.92) | 2972 (3.92) | 8 (5.63) |  |  |  |  |
| Unknown | 20025 (26.33) | 19999 (26.35) | 26 (18.31) |  |  |  |  |
| AJCC N status, n (%) |  |  |  | χ^2^=9.613 | 0.008 |  |  |
| N0 | 50936 (66.98) | 50829 (66.97) | 107 (75.35) |  |  |  |  |
| N1 | 5466 (7.19) | 5452 (7.18) | 14 (9.86) |  |  |  |  |
| Unknown | 19641 (25.83) | 19620 (25.85) | 21 (14.79) |  |  |  |  |
| AJCC M status, n (%) |  |  |  | χ^2^=8.852 | 0.012 |  |  |
| M0 | 49961 (65.70) | 49856 (65.69) | 105 (73.94) |  |  |  |  |
| M1 | 10370 (13.64) | 10348 (13.63) | 22 (15.49) |  |  |  |  |
| Unknown | 15712 (20.66) | 15697 (20.68) | 15 (10.56) |  |  |  |  |
| SEER historic historic stage, n (%) |  |  |  | χ^2^=8.926 | 0.030 |  |  |
| Distant | 10528 (13.84) | 10503 (13.84) | 25 (17.61) |  |  |  |  |
| Localized | 32640 (42.92) | 32567 (42.91) | 73 (51.41) |  |  |  |  |
| Regional | 18295 (24.06) | 18269 (24.07) | 26 (18.31) |  |  |  |  |
| Unknown | 14580 (19.17) | 14562 (19.19) | 18 (12.68) |  |  |  |  |
| Survival month, M (Q_1_,Q_3_) | 10.00 (3.00,26.00) | 10.00 (3.00,25.00) | 14.00 (7.00,39.00) | Z=4.195 | <0.001 |  |  |
| Vital status, n (%) |  |  |  | χ^2^=2.796 | 0.094 |  |  |
| Alive | 19447 (25.57) | 19402 (25.56) | 45 (31.69) |  |  |  |  |
| Dead | 56596 (74.43) | 56499 (74.44) | 97 (68.31) |  |  |  |  |
| SEER cause death classification, n (%) |  |  |  | χ^2^=14.998 | <0.001 |  |  |
| Alive | 19447 (25.57) | 19402 (25.56) | 45 (31.69) |  |  |  |  |
| Death due to liver cancer | 43299 (56.94) | 43240 (56.97) | 59 (41.55) |  |  |  |  |
| Death due to other cause | 13297 (17.49) | 13259 (17.47) | 38 (26.76) |  |  |  |  |

Abbreviations: PSM, Propensity Score Matching; AJCC, American Joint Committee on Cancer; SEER, the Surveillance, Epidemiology, and End Results.

**Supplementary Table 3 The** **effect of** **thyroid cancer history on the risk of** **PLC-specific death among** **PLC patients before PSM matching processing**

| **Variable** | **Model 1** | | **Model 2** | | | | **Model 3** | |  |
| --- | --- | --- | --- | --- | --- | --- | --- | --- | --- |
|  | **HR (95%CI)** | ***P-*value** | | **HR (95%CI)** | ***P-*value** | **HR (95%CI)** | | ***P-*value** | |
| Thyroid cancer history | 0.592 (0.465-0.753) | <0.001 | | 0.583 (0.458-0.744) | <0.001 | 0.618 (0.477-0.801) | | <0.001 | |

Abbreviations: PSM, Propensity Score Matching; PLC, primary liver cancer; HR, hazard ratio; CI, confidence interval.

^a^ Model 1 was established as a crude model without adjustment.

^b^ Model 2 adjusted for age and gender.

^c^ Model 3 adjusted for age, gender, race, marital status, tumor size, regional nodes positive, primary site, histology groupings, surgery, chemotherapy, histologic grade, AJCC T status, AJCC N status, AJCC M status, SEER historic stage.
